# In-Depth Analysis of Tram Curve Noise Dataset for Rigorous Noise Assessment

**DOI:** 10.1038/s41597-025-04660-2

**Published:** 2025-02-22

**Authors:** Sakdirat Kaewunruen, Mohamad Ali Ridho Bin Khairul Anuar, Junhui Huang, Hao Liu

**Affiliations:** University of Birmingham, Birmingham, United Kingdom

**Keywords:** Civil engineering, Mechanical engineering, Developing world

## Abstract

The growth of cities, both socially and economically, has resulted in a higher utilization of trains and high-density mobility. However, this has also led to an escalation of urban environmental issues, specifically pertaining to noise generated by rail operations. The amplified contact between wheels and rails, as well as the vibrations in trains and track structures, contributes to railway noise pollution. While there is existing research on railway noise, there remains a notable gap in the investigation of noise generated specifically at curved sections of railway tracks. Hence, this paper presents 110 sets of tram curve noise datasets, recorded with the MOTIV APP in accordance with ISO 3095:2013 standards. The dataset covers impact, rolling, flanging, and squeal noise, alongside parameters such as speed, direction, crowd levels, and weather conditions. The data are collected by recording the noise generated by the trams at the centre of the curve alignment when the trams traverse along the curve alignment. The comprehensive dataset and analysis contribute valuable insights into tram curve noise, aiding urban planning and noise mitigation efforts.

## Background & Summary

The urbanization trend has led to a significant increase in the use of railways^1,2^, resulting in noteworthy environmental challenges, particularly the escalating noise associated with train operations^3^. As train speeds increase, there is a simultaneous intensification of wheel-rail contact^4^, track structure vibrations^5^, and subsequent railway noise pollution, all of which distinctly impact the well-being of urban residents^6–8^.

The widespread expansion of railway networks has prompted increased scrutiny of associated vibration and noise issues, earning recognition as one of the seven major environmental hazards globally^9,10^. Beyond the immediate disruptions to daily routines and sleep patterns, the adverse effects of traffic noise extend to compromising public health^11–13^.

In a comprehensive investigation of railway noise generation on curves, a study conducted in France revealed key insights. The researchers studied the impact of various factors including train speed, impulse angle, and contact position on noise levels. As a result, the researchers deduced a predictive formula for estimating curved rail noise^14^. However, the study fell short of providing a practical solution to mitigate this specific noise challenge.

Subsequently, Monk-Steel *et al*.^15^ of the Vibration and Noise Research Institute (VNRI) in the UK investigated the influence of transverse creep-slip coefficients and contact-area attachment coefficients on curved rail noise. While they identified correlations between these factors and noise levels, their analysis concentrated narrowly on these parameters, necessitating further exploration of additional factors like train characteristics, track conditions, and ambient surroundings^15^.

In a study investigating noise control and prediction for high-speed trains operating at speeds between 250 and 400 km/h, the researchers conducted comprehensive research where they addressed the challenges associated with noise generated by trains at these elevated speeds. Their research focused on the methodology and experimental data used to calculate noise characteristics generated by high-speed trains. The study also discussed various measures implemented globally to minimize noise from high-speed trains, including local noise barriers and aerodynamic rolling stock design^16^.

In a related work, the evolution of noise and vibration in railways, along with prospective control measures employing mathematical models was studied by Thompson *et al*.^17^. They investigated the evolution of noise and vibration in railways, examining prospective control measures using mathematical models. However, the researchers pointed out that there has been notably less research into curve squeal or impact noise^17^. Another group of researchers investigated the noise transmission and ground vibration resulting from wheel-rail contact, as well as the methods for mitigation^18^. They highlighted the potential impact of ground vibrations from passing trains on nearby residences and structures^19^.

Despite advancements in comprehending railway noise, a substantial knowledge gap persists, particularly concerning the intricacies of noise generated on railway curves. The complexity of curve noise involves a myriad of variables, including vehicle speed, weather conditions, train direction, and crowd density^20^.This complexity underscores the need for more extensive research to unravel the nuanced factors contributing to curve noise^21^. Notably, existing studies on curve noise appear sporadic and region-specific. While comprehensive data on curve noise is accessible for France, limitations in knowledge regarding diverse railway designs and climatic variables across different geographical contexts may impede a thorough understanding of this acoustic phenomenon.

Addressing this gap, this study presents a dataset consisting of 110 sets of tram curve noise recordings, meticulously collected using the MOTIV APP and adhering to ISO 3095:2013 standards. The dataset addresses a significant gap in the field by encompassing diverse noise categories, including impact, rolling, flanging, and squeal noise. These recordings, sampled at a rate of 48 kHz, are strategically conducted at the centre of the curve alignment to capture the nuanced noise generated as trams traverse along the curve alignment.

**Fig. 1:**
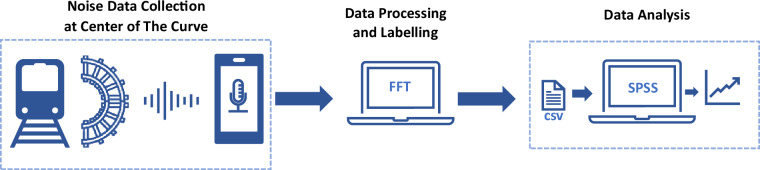
Framework of tram curve noise data collection and analysis.

The dataset comprises 110 raw audio files in WAV format, each with an average duration of 12 seconds. Additionally, a corresponding comma-separated values (CSV) file is created, containing extracted and processed data from the 110 audio files. The processed data, obtained through Fast Fourier Transform (FFT), is presented in decibel format and includes specific categories such as Impact noise, Rolling noise, Flanging noise, and Squeal noise. Figure 1 illustrates the overview of the framework of the data collection.

Furthermore, this CSV file incorporates various contextual parameters, adding depth to the dataset. These parameters include tram speed, direction, crowd levels, and prevailing weather conditions. Crowd levels are classified as either crowded, overcrowded, or uncrowded, providing insight into the potential impact of passenger density on tram noise. Weather conditions are documented as cloudy, sunny, or experiencing light rain, capturing the potential influence of atmospheric factors on tram noise dynamics.

This dataset serves as a valuable resource for researchers as it includes diverse tram noise categories data and provides a nuanced understanding of the acoustic environment during tram operations on curved alignments. The raw audio files and processed data offer a comprehensive foundation for further investigations and analyses in the domain of railway transportation noise research.

## Methods

### Data collection procedures

The recording of tram curve noise is carried out at Birmingham New Street station, focusing on the Caf Urbos 3 tram, which is equipped with a whistling imitation system and a braking system with integral wheels (700 mm diameter). Data is collected over a 43-meter stretch around a tight bend, covering both directions of the track^22^. The MOTIV audio application on a smartphone is utilized for recording, with settings for ‘Audio’ or ‘Sample Rate’ adjusted to ensure compliance with ISO 3095:2013 requirements. The microphone placement followed ISO 3095:2013 standards, with the microphone positioned 7.5 meters horizontally from the centre of the nearest track and at a height of 1.2 meters above the ground. Although the device used was not a Class 1 sound level meter, the setup aligns with ISO 3095:2013 requirements for sample rate and methodology, with recordings made at a 48 kHz sampling rate^23^. To ensure consistency in the measurements, a calibration process was conducted using a standard acoustic calibrator. The calibrator generated a reference sound pressure level (e.g., 94 dB SPL at 1 kHz), which was applied to the microphone prior to recording. The MOTIV Audio app’s input levels were adjusted to match the calibrator’s output, ensuring that the recorded levels accurately represented the sound pressure levels in the environment. This calibration process was repeated after measurements to confirm that no drift occurred during recording.

Noise recordings are undertaken at a specific location (at the centre of the curve alignment) across two days, resulting in 110 sets of full-scale field data. This dataset is significantly larger and more diverse than previous studies, which generally involve only 5–10 pass-by measurements^24^.

**Fig. 2:**
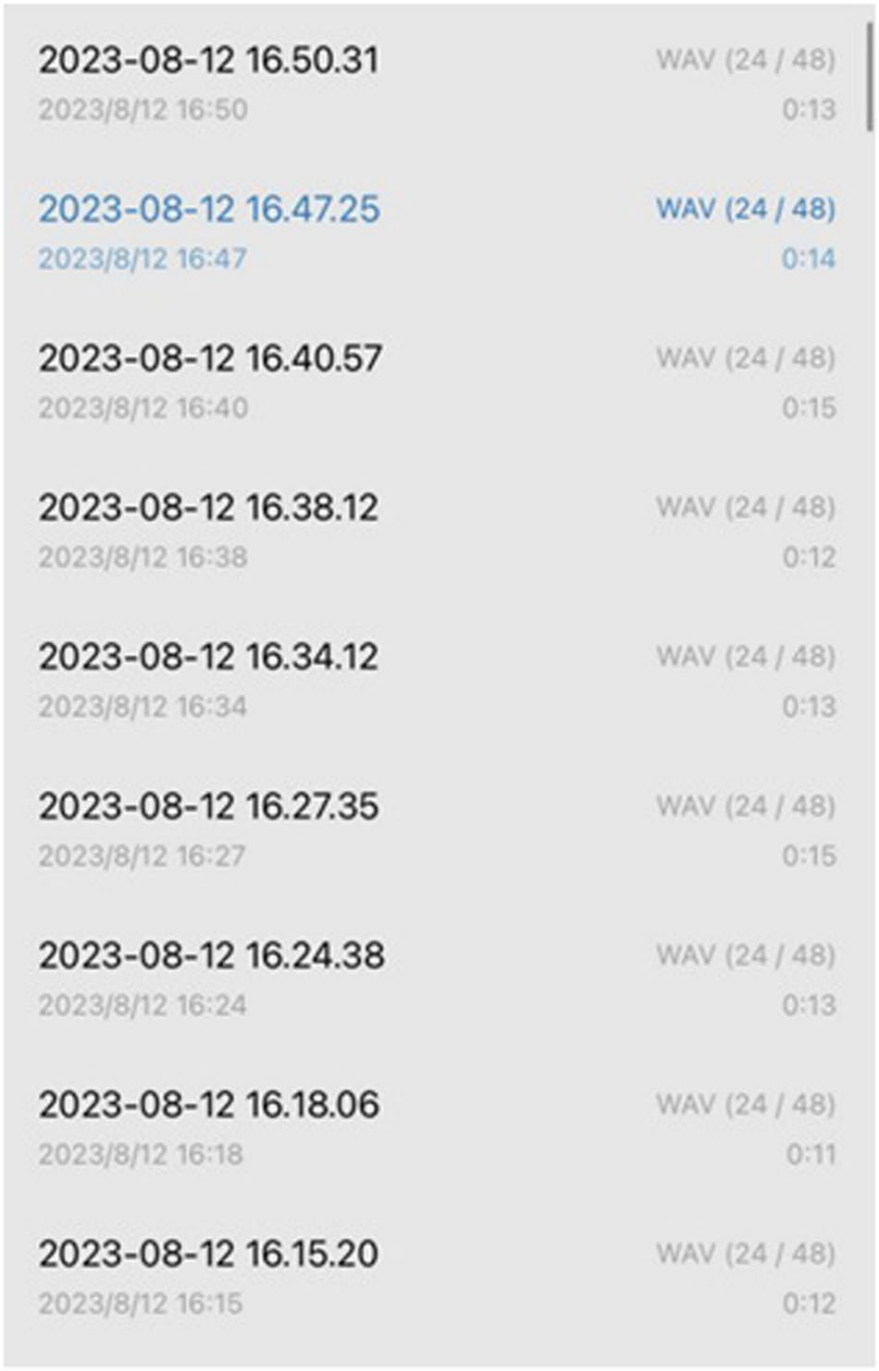
Overview of tram noise audio recordings using the MOTIV audio application.

The tram noise recordings are saved in the Waveform Audio File Format (WAV), with each file having an average duration of 12 seconds. Figure 2 provides an overview of the recorded noise audio. Importantly, MOTIV Audio captures noise from two trams on a specific road section, recording the date and time concurrently. The raw WAV audio files are subsequently processed for detailed analysis.

For the decibel (dB) measurements, the standard reference level employed is 20 micropascals (μPa), which is in alignment with the sound measurement practices outlined in ISO 3095:2013. This level is recognized as the threshold of hearing for an average young adult, forming the basis for expressing sound pressure levels in decibels^23^.

During the data collection process, the crowd level within the tram is systematically recorded, classifying it into distinct categories such as crowded, overcrowded, or uncrowded. This classification aligns with the criteria established by the United Kingdom Rail Safety and Standards Board (UKRSSB)^25,26^.

**Fig. 3:**
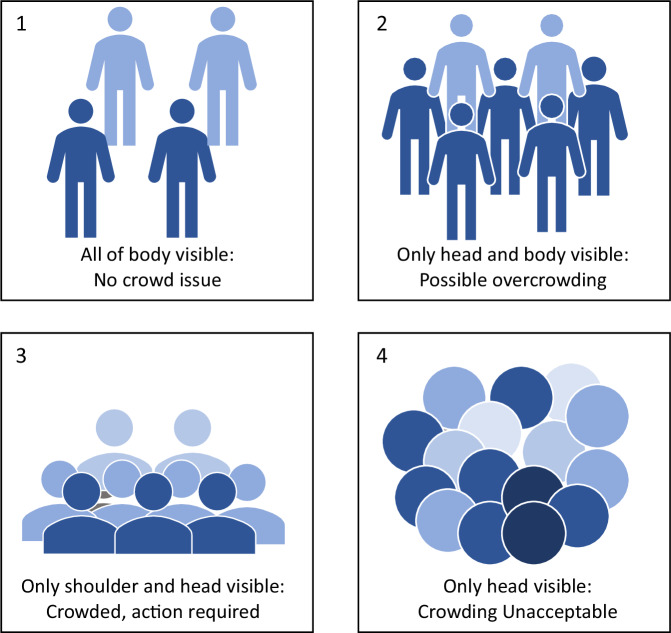
Visual depictions of crowd level in a public transport^25^.

The categorization process involves a meticulous assessment based on visual cues, incorporating specific standards outlined by the United Kingdom Rail Safety and Standards Board (UKRSSB). Continuously monitoring the crowd level within the tram, specific criteria are employed to determine the appropriate classification, as shown in Fig. 3. If the entire body of a person is visible, indicating an absence of crowd issues, this leads to the classification of “uncrowded.”

Conversely, scenarios where only the head and body are visible suggest a potential for overcrowding, prompting the classification as “crowded.” In cases where only the shoulder and head are visible, this indicates a crowded condition requiring immediate attention and intervention.

Adhering to these visual indicators and criteria ensures a consistent and standardized approach to categorizing crowd levels within the tram. Weather conditions such as sunny, light rain, or cloudy are recorded using the built-in weather app on a smartphone, providing real-time environmental data. While this approach captures basic environmental contexts, detailed meteorological data, such as temperature and humidity, were not recorded in this study. Future work will address this limitation by including granular weather data, which could provide deeper insights into the relationship between environmental factors and tram noise. The direction of the tram, encompassing both negative and positive orientations, is also documented throughout the data collection process.

### Data processing procedures

The processing of tram noise audio data is conducted using the Python programming language, where functions are implemented by leveraging several selected Python libraries. The analysis begins with a time-domain examination of the audio signal, focusing on how pressure varies over time. This is visualized through a graph, offering insights into the temporal dynamics of the auditory content. The investigation then transitions to the frequency domain using the Fast Fourier Transform (FFT), a powerful mathematical algorithm widely applied in signal processing. By converting the time-domain signal into its frequency-domain representation, the FFT enables the decomposition of the audio signal into its constituent frequency components^27,28^.

Also, the tram noise data were processed using raw Fast Fourier Transform (FFT) magnitudes to analyse the frequency-domain characteristics of the signals. To reduce spectral leakage, a Hanning window was applied to the time-domain signals before performing the FFT. The Hanning window function is commonly used in spectral analysis to smooth the edges of the signal, ensuring a more accurate frequency representation when transformed to the frequency domain. This approach helps mitigate the effects of discontinuities at the boundaries of finite-length signals. Unlike Power Spectral Density (PSD) estimation methods, which also involve averaging and overlapping windows, the raw FFT magnitudes were used in this study to focus on identifying key frequency peaks and categorizing noise types based on their spectral features.

This frequency-domain analysis provides a detailed view of the amplitude and phase distribution across various frequencies, offering valuable insights into the characteristics of tram noise. Specific noise features, such as impact, rolling, flanging, and squeal, are meticulously examined, leveraging the FFT to isolate and identify prominent frequencies. Python programming is used to implement the analysis, ensuring transparency and reproducibility through a publicly available codebase.

In the context of railway curve noise, the FFT plays a crucial role by enabling the identification of key frequency components essential for characterizing the noise profile. The resulting frequency spectrum, visualized through detailed graphs, highlights the intensity and amplitude of each frequency. These visualizations are instrumental in pinpointing prominent frequencies and assessing the overall noise composition, facilitating a comprehensive understanding of the auditory environment.

It is essential to underscore the significance of selecting specific frequencies for investigation. The systematic examination and identification of these frequencies, made possible through FFT analysis, contribute significantly to understanding the sources and mechanisms underlying tram noise.

A pivotal step in the analytical pipeline involves the implementation of a noise filtering mechanism. This is achieved by selectively zeroing out high-frequency components that are deemed indicative of undesirable noise. The efficacy of this process is evaluated either by playing the filtered audio or persisting it to a new file for subsequent examination.

To augment the interpretability of the findings, amplitude values are converted to the logarithmic decibel (dB) scale, based on the standard reference level of 20 micropascals (μPa). This logarithmic transformation enables a more intuitive comprehension of the amplitude variations present within the audio signal, as changes in decibels provide a clearer perspective on relative loudness and sound pressure levels.

In the subsequent steps, prominent peaks within the frequency domain are identified and isolated, corresponding to distinct noise types such as impact, rolling, flanging, and squeal. These peaks are discerned within frequency boundaries set in alignment with ISO 3095:2013. The frequency range spans from the lower boundary of 20 Hz, which is the lower limit of human hearing, to the upper boundary of 20 kHz, the upper limit of the average human auditory range. This selection ensures comprehensive coverage of all relevant frequencies for tram operations. Visualization of these peaks through graphical representation provides a nuanced understanding of their frequency-specific characteristics within these defined boundaries.

Further analysis involves extracting dB values corresponding to the identified peaks, adhering to sound pressure boundaries also guided by ISO 3095:2013. The lower boundary for sound pressure is set at the threshold of human hearing, approximately 0 dB (20 μPa), while the upper boundary encompasses the maximum levels typically encountered in urban tram environments. This facilitates a detailed quantification of their amplitude characteristics within these sound pressure limits. For enhanced clarity and systematic record-keeping, these findings can optionally be catalogued and stored in Excel files, with each noise type allocated to a dedicated sheet. These sheets are clearly demarcated by the predefined frequency and sound pressure limits as per ISO standards, ensuring a thorough and targeted analysis aligned with urban noise management requirements.

The analysis of tram noise was implemented using Python, leveraging custom functions to systematically identify, quantify, and differentiate noise types, including impact, rolling, squeal, and flanging. The process began with Fast Fourier Transform (FFT) to decompose time-domain audio signals into frequency-domain components. Predefined frequency boundaries were used to isolate each noise types. For instance, impact noise (500–1000 Hz)^5^, rolling noise (centred around 700 Hz)^2^, squeal noise (centred around 700 Hz)^2^, and flanging noise (centred around 500 Hz)^22^. These boundaries ensured that each noise type was analysed independently. A peak detection process, implemented through the ‘get_peaks’ function, further refined this analysis by identifying prominent peaks within the specified frequency ranges.

**Fig. 4:**
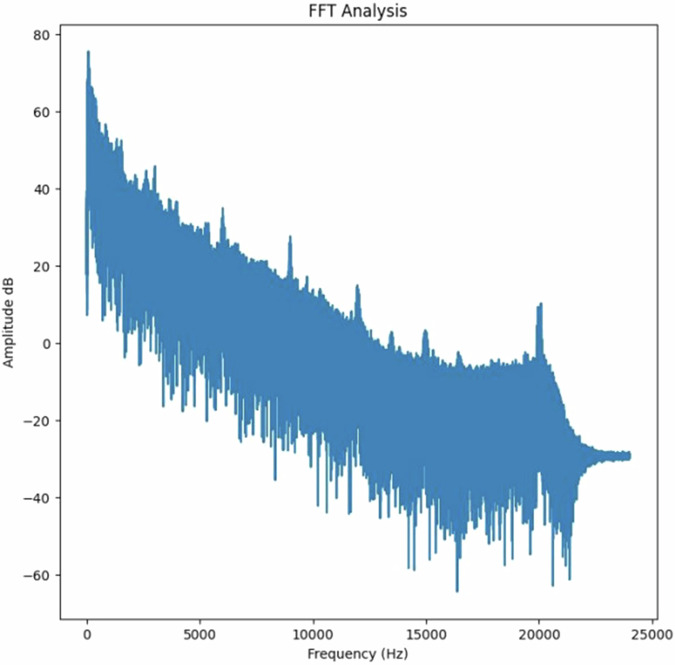
FFT frequency spectrum analysis.

To quantify the noise, the root mean square (RMS) of each peak was calculated, and the amplitudes were optionally converted to decibels (dB) using the ‘convert_db’ function. This allowed for a standardized representation of sound intensities. Noise separation was performed by filtering FFT results, as shown in Fig. 4, within the predefined frequency ranges, followed by reconstruction of filtered signals using inverse FFT. The presence of voices and wind in the sound clips was reduced through frequency-based filtering. The python script provided uses a threshold filter to remove all frequency components below 500 Hz. Frequencies below 500 Hz are commonly associated with wind noise and the lower fundamental frequencies of human voices^29^. By setting these components to zero in the FFT (frequency domain), the script effectively eliminates their influence on the recorded levels. Additionally, the code isolates and analyses specific noise types (impact, rolling, squeal, and flanging) using predefined frequency boundaries, ensuring that irrelevant sounds such as voices and wind are excluded from the analysis. The processed signals were saved as separate audio files for validation.

Batch processing was streamlined using iterative loops that processed multiple audio files simultaneously. Results, including frequency peaks, RMS values, and associated noise types, were compiled into a structured CSV file for systematic record-keeping. This scalable approach ensured efficient analysis of large datasets while maintaining data integrity. This functionality empowers the simultaneous analysis of multiple audio files, obviating the need for manual intervention. Parameters such as input and output directories, as well as a frequency threshold for noise filtering, can be tailored to accommodate specific analytical requirements.

**Fig. 5:**
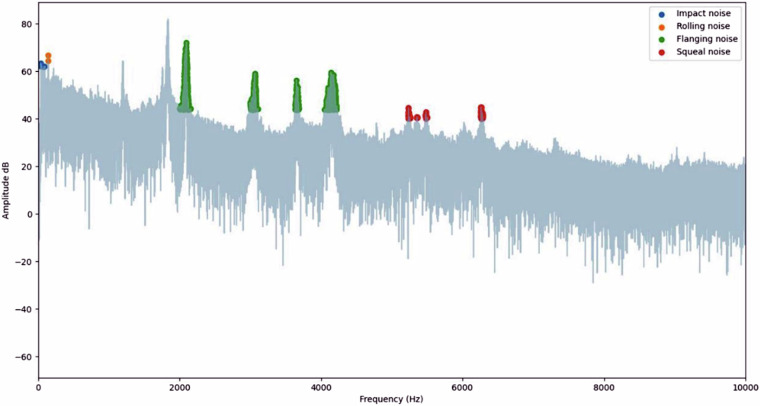
Four types of noise extracted.

This data processing procedures can be regenerate using the python script provided in the repository. By providing these resources, we aim to contribute to the open and collaborative nature of scientific research, promoting the verification and extension of our findings within the academic community. The FFT’s central role in this process highlights its significance in extracting meaningful insights as shown in Fig. 5.

Additionally, tram speeds were recorded as the trams traversed along the curve sections, ensuring a comprehensive dataset for nuanced noise analysis.

The tram speed was determined by calculating the pass-by time, which corresponded to the duration of the recording, and specifically considering a 43-meter stretch on the curved track.

This computation involved dividing the length of the designated curve track by the pass-by time, measured on a per-audio basis. The resulting velocity, expressed in meters per second, provided insights into the tram’s motion dynamics during its traversal along the specified curved trajectory^30^.

Mathematically, the velocity (v) of the tram can be determined using the equation of velocity:

$$\text{v}\,=\,\frac{d}{t}$$

where:

- v represents the velocity in meters per second,
- d denotes the distance covered along the 43-meter stretch of the tight bend
- t signifies the duration of the recording.

The calculated speed of the tram then is tabulated in the CSV file along with the other data such as impact noise, rolling noise, flanging noise, squeal noise, crowd level, weather, and directions of the trams.

### Final dataset

The dataset consists of 110 processed WAV audio files, providing information of tram noise along curved tracks. Distinct noise types, including impact, rolling, flanging, and squeal, are quantified in decibel units.

Additionally, the dataset incorporates contextual variables such as tram speed (m/s), crowd density (categorized as crowded, overcrowded and uncrowded), direction (positive or negative), date, time, and weather conditions (sunny, light rain, or cloudy).

The data as shown in Table 1, is conveniently structured in CSV format for straightforward accessibility.

**Table 1:** Overview of the processed dataset.

Date and Time	Impact Noise (dB)	Rolling Noise (dB)	Flanging Noise (dB)	Squeal Noise (dB)	Speed (m/s)	Direction	Crowd Level	Weather
2023/08/07 18.14.49	40.173	40.087	19.357	6.718	3.071	negative	uncrowded	sunny
2023/08/07 18.18.12	31.236	29.869	34.344	8.649	3.071	Positive	uncrowded	sunny
2023/08/07 18.23.45	31.545	32.431	23.876	6.382	2.867	negative	overcrowded	sunny

This dataset’s profound relevance lies in its ability to provide researchers with a nuanced understanding of the acoustic intricacies specific to curve sections. The inclusion of various contextual parameters enriches the dataset, allowing researchers to discern patterns and correlations between tram operations and the diverse noise profiles observed during curved track traversals. Such insights are invaluable for formulating targeted noise mitigation strategies, tailoring interventions to the distinctive challenges posed by curve-induced noise.

This dataset holds promising applications in predictive modelling, presenting an opportunity for the development of advanced machine learning algorithms. By training algorithms on the diverse dataset, there exists the potential to predict noise levels in curve sections by considering a blend of operational and environmental variables. This predictive capacity becomes crucial for proactive noise management, allowing for the implementation of pre-emptive measures aimed at minimizing noise impact specifically in regions marked by curved track configurations.

In essence, the dataset’s future utility lies in its capacity to inform and guide strategic noise mitigation efforts, fostering a proactive and targeted approach in addressing the acoustic challenges associated with tram operations along curved tracks.

Researchers can leverage this dataset to assess the performance of existing solutions and, based on the detailed documentation, refine or develop novel technologies tailored to address the unique challenges posed by curve-induced noise.

This iterative process of assessment and refinement contributes to the ongoing optimization of noise mitigation strategies, ultimately fostering quieter and more sustainable railway operations. In the broader context of urban soundscape research, the dataset offers a comprehensive foundation for understanding how curve-induced noise integrates into the larger acoustic environment.

Researchers can utilize this dataset for in-depth studies, tracking the evolution of noise patterns over time. This enables the identification of trends and assessment of the impact of infrastructure changes or noise reduction initiatives. The dataset provides valuable context, including information on tram speed, crowd density, direction, and weather conditions.

In summary, systematically documented dataset, emerges as a cornerstone for progressing the exploration of railway noise, especially in curve sections. From enhancing our understanding of acoustic nuances to facilitating the development of predictive models and noise reduction technologies, this dataset paves the way for a comprehensive and sustainable approach to managing the acoustic footprint of railways in urban environments.

## Data Records

The dataset is uploaded at URL https://zenodo.org/doi/10.5281/zenodo.10975444^31^, alongside with three main folders: ‘Tram Curve Noise TBD,’ ‘Python Script TBD,’ and ‘Processed Data TBD.’ The ‘Tram Curve Noise TBD’ folder contains around 196 megabytes (MB) of audio files in WAV format^32^.

In the https://zenodo.org/records/10975445 folder, you’ll find CSV files with features extracted using Fast Fourier Transform (FFT) analysis. These features highlight various noise components like impact, rolling, flanging, and squeal. The dataset also incorporates additional details such as tram speed, direction, crowd level, and weather conditions, contributing to its comprehensive nature. Table 1 shows a concise overview of the processed dataset.

To facilitate easy reproduction of our work, we provide the Python Script in the folder, which includes a Python script. This script encompasses our entire analytical process, involving raw FFT analysis, noise feature extraction, and other relevant steps. Researchers interested in understanding and replicating our findings can use this Python script, promoting transparency and collaboration in the scientific community.

## Technical Validation

### Audio

The recording was conducted at Birmingham New Street station, focusing on a Caf Urbos 3 tram model equipped with a whistling imitation system and a braking mechanism featuring integral wheels (700 mm diameter). Data collection took place at the centre of the curved track. The microphone placement adhered to ISO 3095:2013 standards, with the microphone positioned 7.5 meters horizontally from the centre of the nearest track and at a height of 1.2 meters above the ground. The ‘Audio’ and ‘Sample Rate’ settings on the app were configured to comply with ISO 3095:2013 standards, ensuring that all collected data met internationally recognized parameters.

### Data pre-processing

Each phase of the audio preparation undergoes a meticulous manual qualitative review, involving an attentive listening process to ensure the accuracy and quality of the data. Using the Python programming language and raw FFT, the analysis explores both time and frequency domain characteristics, which are essential for understanding the complex dynamics of tram noise. The FFT, a powerful signal processing tool, plays a critical role in identifying and isolating frequencies that define various noise aspects, particularly useful in analysing railway curve noise. Predefined frequency boundaries are applied to isolate distinct noise types, such as impact, rolling, squeal, and flanging, ensuring that each noise type is analysed independently.

To quantify the noise, the root mean square (RMS) of each frequency peak is calculated, and amplitudes are optionally converted to decibels (dB) for a standardized representation of sound intensity. The data is further refined through a noise filtering mechanism that selectively removes undesirable high-frequency components, ensuring data integrity. A threshold filter is applied to eliminate frequency components below 500 Hz, which are often associated with wind noise and the fundamental frequencies of human voices. This helps minimize the influence of irrelevant sounds on the recorded levels. Following this, inverse FFT is applied to reconstruct the filtered signals.

A robust validation process is employed, combining both qualitative and quantitative evaluations. The identification and isolation of prominent peaks within the frequency domain further refine the analysis, with each peak corresponding to a distinct noise type. This comprehensive approach ensures that the tram noise dataset analysis is precise, reliable, and transparent, aligning with internationally recognized standards, such as ISO 3095:2013. Batch processing is streamlined using iterative loops that allow for the efficient analysis of large datasets, and results, including frequency peaks, RMS values, and corresponding noise types, are organized in a structured CSV file for systematic record-keeping. This thorough methodology guarantees that the tram curve noise analysis is performed with technical rigor and transparency.

### Bayesian analysis

The processed data is loaded into the Statistical Package for the Social Sciences (SPSS) software for further statistical analysis. For this study, Bayesian analysis is chosen because its results typically describe the posterior distribution, representing parameter uncertainty based on observed data^33^. Interpreting these results involves understanding the posterior distribution and, in some cases, evaluating Bayes factors^31^. Also, Bayesian analysis was employed to explore the probabilistic relationships between tram operational factors (speed, direction, crowd levels, and weather conditions) and noise characteristics (rolling, impact, flanging, and squeal noise). This approach provides a robust framework for quantifying uncertainties in predictor effects, enabling a deeper understanding of the intricate dynamics shaping tram curve noise. Bayesian analysis was chosen over traditional methods due to its ability to handle variability and produce credible intervals that offer a clearer interpretation of parameter uncertainty.

The dataset undergoes meticulous validation employing Bayesian analysis, specifically evaluating the interconnections among tram curve noise and a range of predictors such as rolling noise, impact noise, flanging noise, squeal noise, crowd level, direction of the tram, weather, and speed of the tram. This rigorous approach contributes significantly to a nuanced understanding of the dataset’s characteristics.

**Fig. 6:**
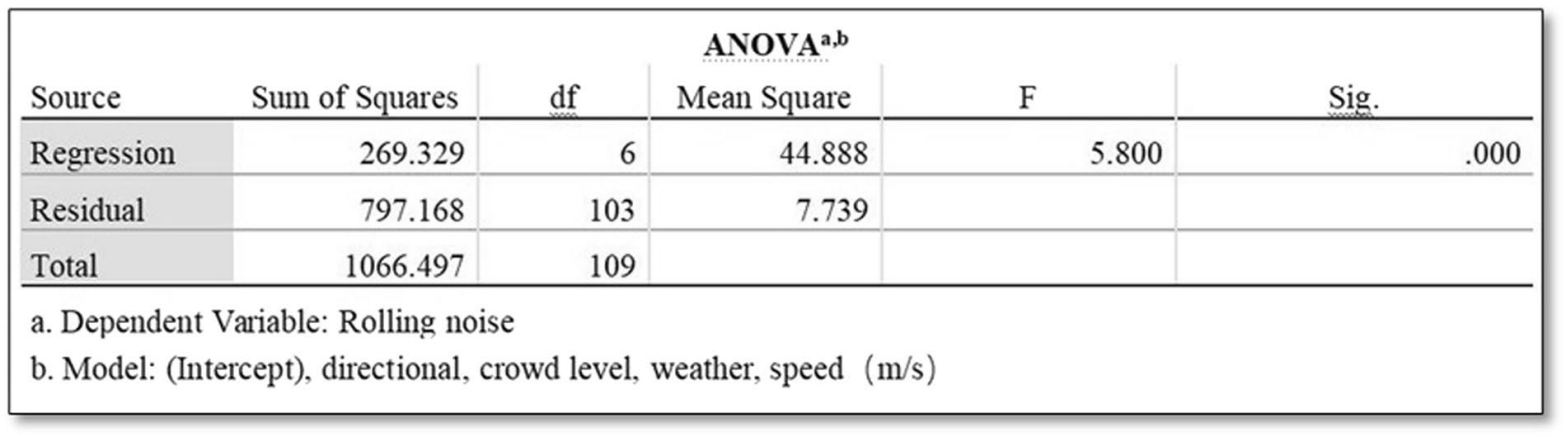
ANOVA for rolling noise.

In Fig. 6, the F statistic, denoted as F = 5.80, serves as a pivotal metric for evaluating the predictive efficacy of the model using the collected dataset. This assessment is made by comparing the model to a benchmark model, which includes solely the constant term and omits the influence of any predictors. An F statistic exceeding 1 indicates an improvement in predictive capabilities relative to the benchmark. The observed F = 5.80 in this context represents a substantively large value, emphasizing that the model, based on the collected dataset, significantly enhances predictive accuracy compared to the benchmark model. This statistical observation underscores the model’s notable prowess in leveraging the dataset to outperform a simplified baseline.

**Fig. 7:**
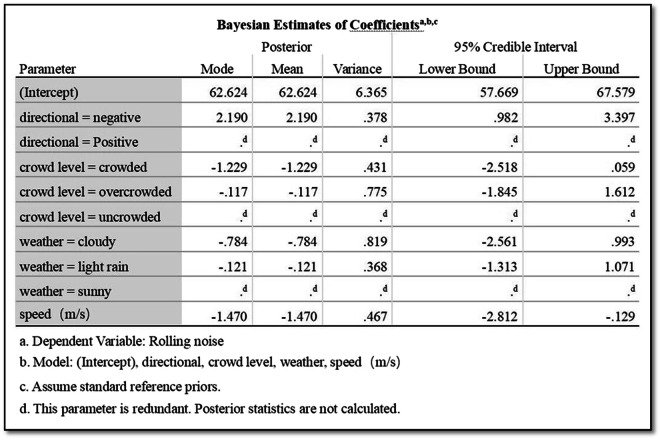
Bayesian estimates of coefficients of rolling noise.

**Fig. 8:**
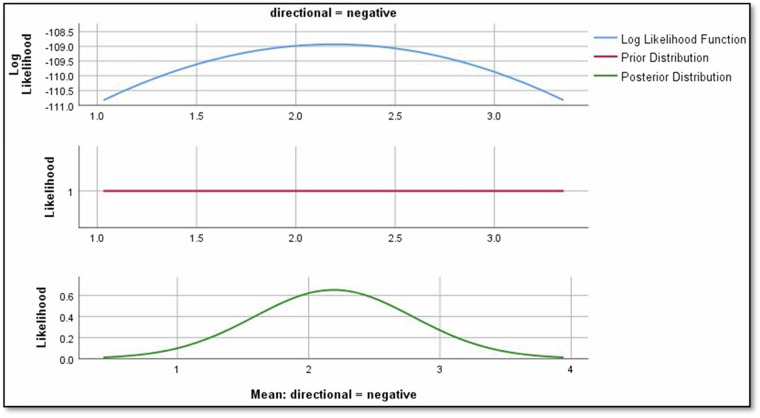
The impact of negative directional on rolling noise.

Figure 7 reveals crucial insights into the impact of predictors on Rolling noise. The mean regression parameters for directional factors, crowd levels, weather conditions, and speed are quantified, providing a nuanced understanding of their respective relationships. The mean value of the regression parameter for directional = negative is 2.190, indicating that directional = negative has a positive effect relationship on Rolling noise as shown in Fig. 8.

**Fig. 9:**
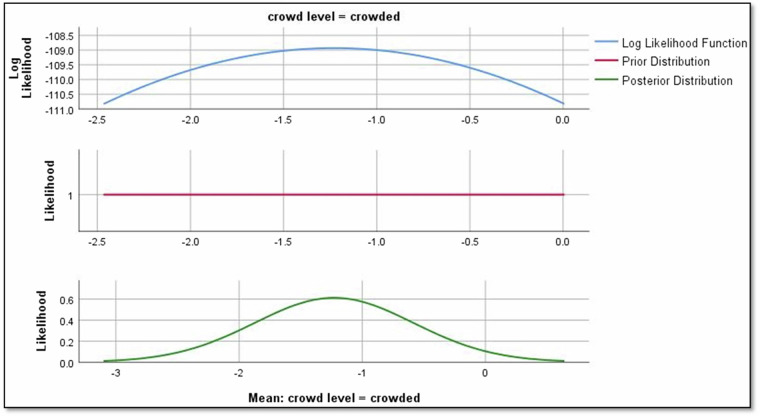
The impact of crowd level in crowded conditions on rolling noise.

**Fig. 10:**
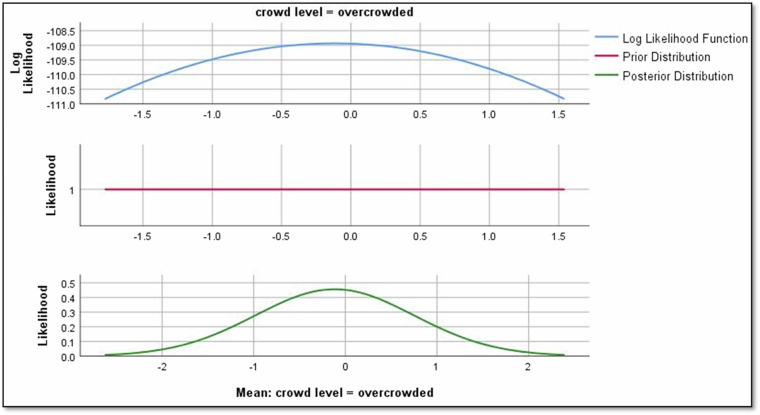
The impact of crowd level in overcrowded conditions on rolling noise.

The analysis of regression parameters reveals valuable insights into the impact of different factors on Rolling noise. For crowd levels, the mean regression parameter for “crowded” is -1.229, indicating a negative effect on the Rolling noise relationship as shown in Fig. 9. Conversely, for “overcrowded,” the mean parameter is −0.117, suggesting a positive effect on Rolling noise as shown in Fig. 10. Notably, a parameter of 0 for “uncrowded” implies no discernible impact on Rolling noise in this context.

**Fig. 11:**
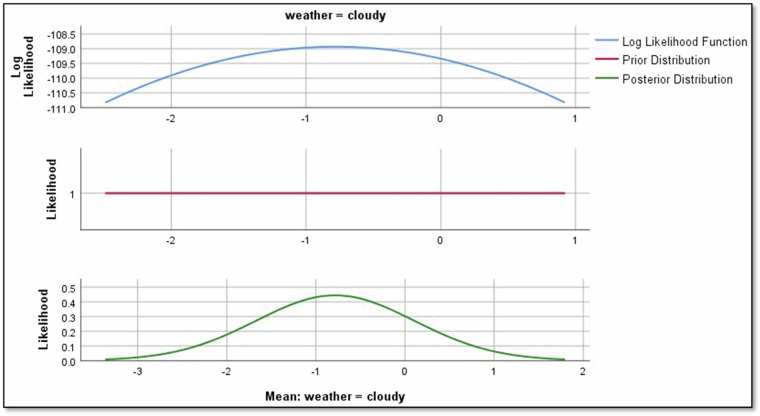
The impact of cloudy weather on rolling noise.

**Fig. 12:**
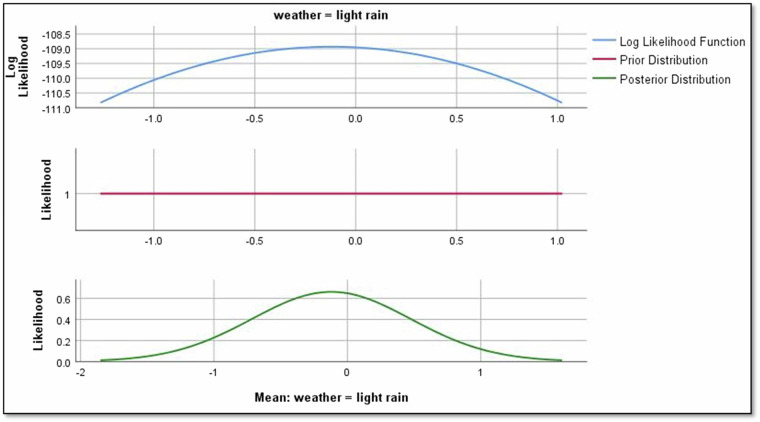
The impact of light rain weather on rolling noise.

Moving to weather conditions, the mean regression parameter for “cloudy” is -0.784, indicating a negative effect on Rolling noise as shown in Fig. 11. Similarly, for “light rain,” the mean parameter is −0.121, signalling a negative influence on Rolling noise as shown in Fig. 12. However, for “sunny” conditions, the regression parameter is 0, suggesting no observable effect on Rolling noise.

**Fig. 13:**
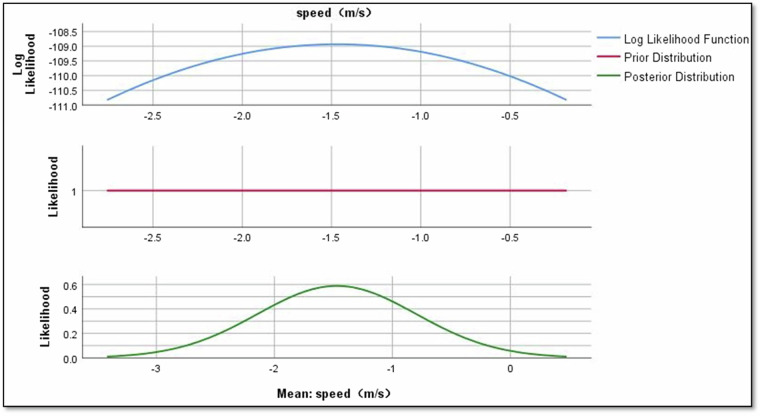
The impact of speed on rolling noise.

In terms of speed, the mean regression parameter is −1.470, pointing to a negative effect on Rolling noise as shown in Fig. 13. This implies that as the speed increases, there is a corresponding decrease in Rolling noise levels. These regression parameters provide a quantitative understanding of the directional impact each variable has on the phenomenon of Rolling noise.

Utilizing Bayesian estimation allowed for a detailed examination of coefficients and error variance. The mean regression parameter values for directional factors, crowd levels, and weather conditions provided insights into their respective impacts on rolling, flanging, impact, and squeal noise.

Results revealed significant relationships between predictors and noise types. For instance, directional factors positively influenced rolling noise, with a mean regression parameter of 2.190 for ‘negative’ direction (Fig. 8). Crowd density also played a nuanced role: while ‘crowded’ conditions reduced rolling noise (−1.229), ‘overcrowded’ conditions slightly increased it (−0.117; Figs. 9,10). Weather conditions showed mixed effects, with ‘cloudy’ and ‘light rain’ reducing rolling noise (−0.784 and −0.121, respectively; Figs. 11,12), while ‘sunny’ conditions had no discernible impact. Bayesian validation, including posterior predictive checks and parameter estimation, confirmed the model’s robustness. Notably, credible intervals for regression parameters consistently captured observed data points, underscoring the reliability of the findings. By leveraging Bayes factors, speed and tram direction were identified as the most influential predictors, guiding future mitigation strategies. The Bayesian framework not only elucidated these relationships but also highlighted areas for further exploration, such as the interplay between crowd density and squeal noise.

The same analysis is carried out to investigate the relationship between the speed of the tram, directional factors, weather conditions, and crowd level with impact noise, squeal noise, and flanging noise. A comprehensive analysis is meticulously conducted to explore the nuanced correlations among tram speed, directional variations, weather conditions, and crowd levels, specifically concerning tram curve noise, impact noise, squeal noise, and flanging noise. This thorough investigation ensures a detailed understanding of the intricate dynamics shaping tram curve noise. Furthermore, the replicability of this analysis is facilitated by the provision of robust datasets, ensuring that the same results can be reproduced and serving as a valuable resource for extended inquiries into the complexities of tram acoustics, particularly in the context of tram curve noise.

## Data Availability

The code and accompanying scripts utilized for the conversion of sound files into a meaningful format have been made publicly accessible through the repository at https://zenodo.org/records/11540120^32^. Crafted with the Python programming language, the code draws on specific libraries to fulfil its tasks. Key dependencies include NumPy for numerical operations, Soundfile (sf) for handling sound files, Sounddevice (sd) for sound playback, Matplotlib.pyplot (plt) for creating visualizations, Pandas (pd) for data manipulation, and SciPy.stats (stats) for statistical analyses. These dependencies collectively empower the code to perform various operations, spanning time and frequency domain analyses, Fast Fourier Transform (FFT) transformations, noise filtering, and visualization of data.
